# Early treatment with laronidase improves clinical outcomes in patients with attenuated MPS I: a retrospective case series analysis of nine sibships

**DOI:** 10.1186/s13023-015-0344-4

**Published:** 2015-10-07

**Authors:** Nouriya A. Al-Sannaa, Luisa Bay, Deborah S. Barbouth, Youssef Benhayoun, Cyril Goizet, Norberto Guelbert, Simon A. Jones, Sandra Obikawa Kyosen, Ana Maria Martins, Chanika Phornphutkul, Celia Reig, Rebecca Pleat, Shari Fallet, Iva Ivanovska Holder

**Affiliations:** Johns Hopkins Aramco Healthcare, Dhahran, Saudi Arabia; Department of Inherited Errors of Metabolism, Hospital Juan P. Garrahan, Buenos Aires, Argentina; Dr. John T. Macdonald Foundation, Department of Human Genetics, University of Miami Miller School of Medicine, Miami, FL USA; Pediatric Services, Robert Boulin Hospital, Libourne, France; CHU Bordeaux, Pellegrin Hospital, Department and Univ. Bordeaux, laboratoire MRGM (EA4576), Bordeaux, France; Metabolic Section, Children’s Hospital of Córdoba, Córdoba, Argentina; Manchester Centre for Genomic Medicine, St. Mary’s Hospital, CMFT, University of Manchester, Manchester, UK; Reference Center for Inborn Errors of Metabolism, Federal University of São Paulo, São Paulo, Brazil; Division of Human Genetics, Department of Pediatrics, Hasbro Children’s Hospital, Brown University, Providence, RI USA; Pediatric Division, General Hospital of Segovia, Segovia, Spain; Genzyme, a Sanofi company, 500 Kendall Street, Cambridge, MA 02142 USA; Pfizer Inc, New York City, NY USA

**Keywords:** Enzyme replacement therapy, Early diagnosis, Early treatment

## Abstract

**Background:**

Enzyme replacement therapy (ERT) with laronidase, (recombinant human α-L-iduronidase; Aldurazyme) is the primary treatment option for patients with attenuated mucopolysaccharidosis type I (MPS I). This study examined the effect of early ERT on clinical manifestations.

**Methods:**

This multinational, retrospective case series abstracted data from records of 20 patients with Hurler-Scheie syndrome within nine sibships that included older siblings treated with laronidase after the development of significant clinical symptoms, and younger siblings treated before significant symptomatology. Median age at diagnosis was 5.6 and 0.5 years for older and younger siblings, respectively. Median age at ERT initiation was 7.9 and 1.9 years for older and younger siblings, respectively.

**Results:**

Improvement or stabilization of somatic signs and symptoms was more notable in younger siblings. Organomegaly present at onset of ERT improved in the majority of both older and younger siblings. Analysis of physician-rated symptom severity demonstrated that cardiac, musculoskeletal, and cognitive symptoms, when absent or mild in younger siblings at ERT initiation, generally did not develop or progress. The majority of older siblings had height/length Z-scores greater than two standard deviations below the mean (less than -2) at both time points. In general, Z-scores for younger siblings were closer to the sex- and age-matched means at follow-up.

**Conclusions:**

These findings suggest early initiation of laronidase, prior to the onset of symptoms in patients with attenuated MPS I, can slow or prevent the development of severe clinical manifestations.

## Background

Mucopolysaccharidosis type I (MPS I) is an autosomal recessive disorder caused by deficient α-L-iduronidase activity, a lysosomal enzyme involved in degradation of the glycosaminoglycans (GAGs) heparan and dermatan sulfate. Enzyme deficiency leads to accumulation of GAGs in multiple tissues resulting in a progressive and multisystem disease. MPS I includes a spectrum of clinical presentations ranging from severe (Hurler syndrome) to attenuated (Hurler-Scheie, [H/S] and Scheie syndromes) [1]. Patients with Hurler syndrome develop coarse facial features, debilitating skeletal disease, organomegaly, cognitive impairment, and cardiac and respiratory disease shortly after birth. Patients with H/S have mild or no cognitive impairment; however somatic symptoms result in significant disease morbidity and reduce life expectancy to the second or third decade of life. Disease progression is slowest in patients affected with Scheie syndrome.

Treatment options include hematopoietic stem cell transplant (HSCT) recommended before 2.5 years of age for patients with the most severe phenotype, and enzyme replacement therapy (ERT) with laronidase (recombinant human α-L-iduronidase; Aldurazyme®) indicated as the primary treatment option for patients with attenuated MPS I [2]. Laronidase administration is safe and effective, and improvements in clinical manifestations and stabilization of disease progression are achieved in patients with varying degrees of disease severity [3–6]. The long-term clinical benefits achieved with ERT appear to be largely dependent on early diagnosis and treatment [7]. Clinical features such as cardiac valve disease, corneal clouding, and skeletal changes do not respond as well to ERT since irreversible pathology may be established by the time symptoms appear [8].

Due to the large phenotypic heterogeneity seen in MPS I, correlating ERT timing to the effect on the clinical course and progression of symptoms can be challenging. However, it is evident from assessing treatment outcomes in siblings with similar genetic backgrounds and expected rates of disease progression that pre-symptomatic initiation of ERT drastically alters the clinical course of MPS I [9, 10]. In two sets of siblings, more serious clinical findings were well documented for older siblings initiating ERT later in life after the onset of symptoms compared with their younger counterparts who commenced treatment before disease manifestations were apparent. In both cases, early ERT prevented the development of many clinical features of MPS I in the younger sibling [9, 10].

Here, we present the largest compilation of sibling data to date for patients with the H/S phenotype. This case series examines data from sibling pairs/sibships that included older siblings treated with laronidase after the development of significant clinical symptoms, and younger siblings, the majority of whom initiated laronidase before significant symptomatology.

## Methods

This multinational, retrospective, chart review case series was conducted at the authors’ institutions. All patients with MPS I diagnosed by enzyme biochemical determination and/or molecular analysis were treated with laronidase according to prescribing information (weekly intravenous infusions of 100 U/kg of body weight). Sibships were identified where younger siblings initiated ERT at an earlier age than older siblings. In general, older siblings initiated treatment with laronidase after the onset of MPS I symptoms while younger siblings initiated ERT before onset of significant physical and clinical symptoms. Written informed consent was provided by the legal guardians/parents of all patients.

Treating physicians completed questionnaires assessing signs and symptoms of MPS I prior to and following ERT. Data from patient records, medical histories and neurological, cardiovascular, pulmonary, gastroenterology, musculoskeletal, ophthalmologic, audiologic, and psychomotor developmental examinations were abstracted including:

Height/length (absolute); Description of facial features observed during physical examination; Presence of corneal opacity, hearing loss/use of hearing aids, sleep apnea, hernia; Results of pulmonary function tests (PFT); Liver and spleen size determined by palpation and/or imaging studies; Cardiac disease description/severity determined from echocardiogram; Joint and skeletal disease observed during physical examination and/or radiographic imaging; Presence/severity of dysostosis multiplex, joint contractures and affected joints; Motor development assessed during physical examination; Cognitive and language development assessed by formal evaluation or by investigator impression; and Ability to perform activities of daily living (ADL).

Data were abstracted at a time point just prior to initiating ERT (Time point 1, T1) and follow-up data were collected after at least 3 years of ERT (Time point 2, T2).

### Data analysis

The frequency of signs and symptoms at T1 was determined from a maximum value of 14 symptoms that included coarse facial features, corneal clouding, hearing loss, sleep apnea, abnormal PFT, cardiac abnormalities, hepatomegaly, splenomegaly, hernia, dysostosis multiplex, joint contractures, motor developmental delay, language/cognitive delay, and restrictions in ADL. Symptom severity was rated by physicians for corneal opacity, cardiac symptoms, dysostosis multiplex, and joint contractures. Z-scores for height/length were calculated according to National Health and Nutrition Examination Survey and Center for Disease Control Growth Chart Information as revised on June 8, 2000.

## Results

Twenty patients within nine sibships (seven sibling pairs and two sets of three siblings) were identified from nine centers in Argentina (two sites), Brazil, France, Spain, Saudi Arabia, UK, and the US (two sites). Median age (range) at diagnosis was 5.6 years (1, 9) for older siblings (*n* = 10) and 0.5 years (0, 6.7) for younger siblings (*n* = 10). All older siblings initiated laronidase by 14 years of age, while younger siblings initiated therapy before 4 years of age (with one exception). Five younger siblings (sibships 1–5a) were diagnosed at less than 1 year of age and initiated treatment by 1.3 years of age. For analysis of responses to treatment, these patients are presented together as Group A. A second group of younger siblings (Group B, sibships 5b–8) began laronidase treatment after age 2. Age at diagnosis, treatment, and duration of treatment for each patient, and median data by group are presented in Table 1. Older and younger siblings within a sibship had similar ERT duration.

**Table 1:** Demographics and baseline characteristics of individual patients

Sibship ID	Pt ID	Sex	Genotype	Age at diagnosis (yr)		Age at ERT Initiation (T1) (yr)		Duration of ERT at T2 (yr)	
Group A^a^									
1	OS	F	P533R/P533R	8		8.8		5.4	
1	YS	M	P533R/P533R	0.2		0.3		4.5	
2	OS	M	P533R/P533R	1.3		3.3		11.7	
2	YS	F	P533R/P533R	Birth		0.4		9.6	
3	OS	F	NA	1		12		8	
3	YS	F	NA	0.4		0.7		8.3	
4	OS	M	G208D/P520R	3.6		5.5		3.5	
4	YS	M	G208D/P520R	0.8		1.3		3.6	
5a	OS	F	L490P/L490P	3		5.8		8.6	
5a	YS1	M	L490P/L490P	0.1		0.3		7.6	
				Group A Median (range)					
				OS	YS	OS	YS	OS	YS
				*N* = 5^c^	*N* = 5	*N* = 5^c^	*N* = 5	*N* = 5^c^	*N* = 5
				3.0	0.2	5.8	0.4	8.0	7.6
				(1, 8)	(0, 0.8)	(3.3, 12)	(0.3, 1.3)	(3.5, 11.7)	(3.6, 9.6)
Group B^b^									
5b^c^	OS	F	L490P/L490P	3		5.8		8.6	
5b	YS2	F	L490P/L490P	0.1		2.5		8.8	
6	OS1	M	NA	9		13		6	
6	OS2	M	NA	8		11		6	
6	YS	M	NA	0.6		3		7	
7	OS	F	P533R/L564fsx	4.3		4.5		5.5	
7	YS	F	P533R/L564fsx	3.3		3.4		5.6	
8	OS	M	NA	6.8		7		6.0	
8	YS	M	NA	3.3		3.7		5.8	
9	OS	F	NA	7		14		7	
9	YS	F	NA	6.7		8.6		6.4	
				Group B Median (range)					
				OS	YS	OS	YS	OS	YS
				*N* = 6	*N* = 5	*N* = 6	*N* = 5	*N* = 6	*N* = 5
				6.9	3.3	9.0	3.4	6.0	6.4
				(3, 9)	(0.1, 6.7)	(4.5, 14)	(2.5, 8.6)	(5.5, 8.6)	(5.6, 8.8)

*F* female, *M* male, *NA* not available, *OS* older sibling, *YS* younger sibling

^a^Group A: younger siblings ERT onset ≤1 year of age

^b^Group B: younger siblings ERT onset between 2 and 4 years (except #9 at 8.6 year)

^c^The older sibling in sibship 5 is listed twice (once in group A and once in Group B) since one of the two younger siblings initiated ERT at 0.3 years of age (sibling 2a, Group A) and the other at 2.5 years of age (sibling 2b, Group B), respectively

**Fig. 1:**
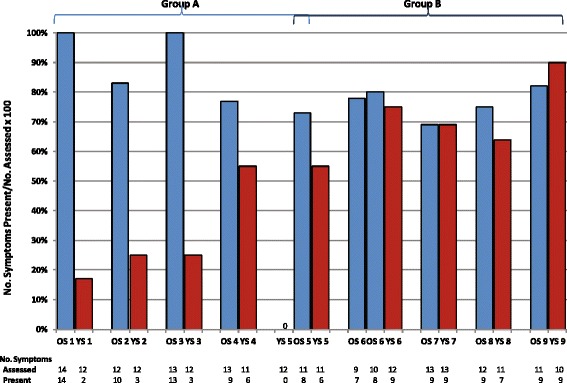
Symptoms present at T1 for Older (OS) and Younger (YS) Siblings. Frequency of signs and symptoms at T1 was determined from a maximum of 14 possible symptoms that included coarse facial features, corneal clouding, hearing loss, sleep apnea, abnormal lung function tests, cardiac abnormalities, hepatomegaly, splenomegaly, hernia, dysostosis multiplex, joint contractures/other skeletal defects, motor developmental delay, language/cognitive delay, and restrictions in ADL

Figure 1 summarizes the percentages of signs and symptoms present at T1 (out of the total number of signs/symptoms assessed per patient) for all patients by sibship. Younger siblings (blue bars) generally had fewer symptoms than older siblings (red bars) at T1, particularly in Group A. All older siblings were symptomatic at T1.

### Treatment outcomes

**Fig. 2:**
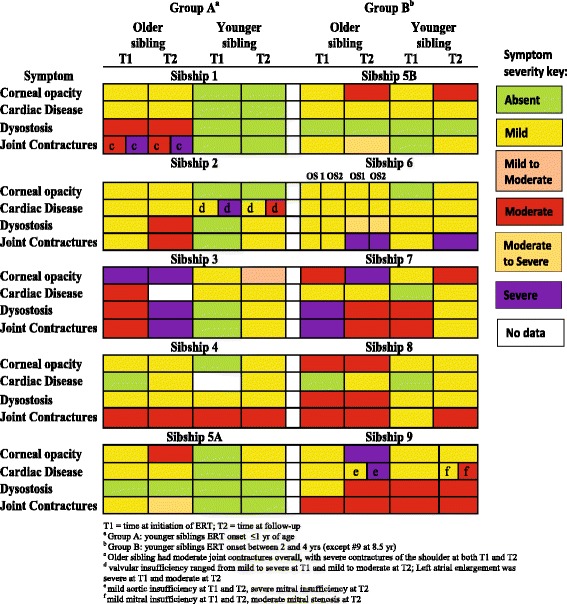
Physician-rated Severity of Symptoms at T1 and T2. Figure 2 Severity of Symptoms at T1 and T2. Severity of key clinical symptoms for older and younger siblings in at the initiation of ERT (T1) and at follow-up (T2) was rated by physicians. Results are organized by group and coded by color. Symptoms were absent, or ranged in severity from mild to severe

Symptom severity as rated by physicians for key clinical symptoms at T1 and T2 is coded by color in Fig. 2. Symptoms were absent or mild for younger siblings in Group A. For younger siblings in Group B, symptom frequency was greater, but motor and cognitive developmental deficits were typically less severe than for older siblings. All patients had improvement or stabilization of some somatic signs and symptoms at the T2 assessment. In general, organomegaly present at ERT onset improved in both older and younger siblings. Earlier treatment with laronidase did not prevent development of corneal clouding in patients asymptomatic at time of treatment. However, analysis of cardiac, musculoskeletal, and motor and cognitive development indicate that younger siblings were less likely to develop or have progression of some symptoms compared to the older siblings in the sibships.

For younger siblings in Group A, who had fewer and more mild symptoms at T1, cardiac and musculoskeletal symptoms generally remained mild or had not developed at the T2 assessment. This was particularly apparent in sibships 1, 3, 4, and 5. For younger siblings in Group B, who had a higher incidence of moderate or severe symptoms at T1, cardiac or musculoskeletal symptoms generally were not as severe and did not progress compared to older siblings.

#### Sibship 1

The younger patient had no major somatic signs or symptoms prior to initiating laronidase and had normal psychomotor development. At T2, after almost 5 years of ERT, there was no development of cardiac or musculoskeletal disease, and except for stabilization of upper airway obstruction and development of mild hernia, the child remains in good health. In contrast, the older sibling already had significant symptoms at 8 years of age (T1). In addition to symptoms noted in Fig. 2, the patient had mild hearing loss and psychomotor delay, speech difficulty, and lung function tests 50 % of normal. The patient required help in all ADL. Symptoms present at T1 were stabilized or improved (organomegaly and hearing) at T2, except for worsening of kyphoscoliosis.

#### Sibship 2

**Fig. 3:**
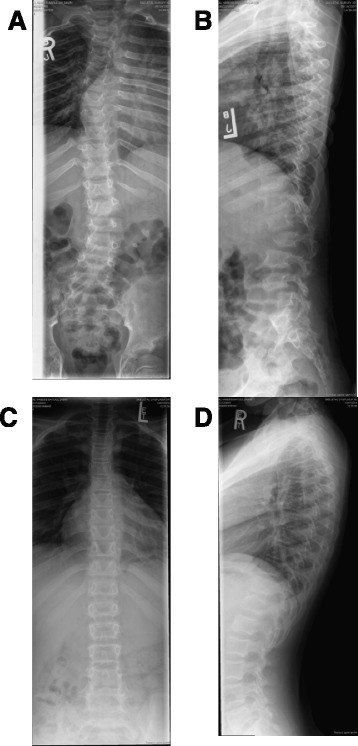
Spinal radiographs from sibship 2 patients. Panel **a** and **b** show radiographs of the older sibling at age 8, after 5 years of ERT. Note the ‘S’ shaped scoliosis with right-sided convexity in the thoracic region and left-sided convexity in the lumbar region as well as gibbus deformity at L1 and L4. There is retrolisthesis of L4 with respect to L5 and possible compromise in the spinal canal at L1 and L4. Small anterior beaking is noted in the lower thoracic and lumbar vertebrae. Panel **c** and **d** are spinal radiographs of the younger sibling at age 10, after 10 years of ERT. Note the absence of spinal deformity with only minimal anterior beaking of the lower thoracolumbar vertebrae

The younger sibling was 5 months of age at T1 and already had cardiac symptoms ranging from mild to severe (Fig. 2), and mild organomegaly. After 10 years of ERT, cardiac symptoms showed some improvement into the mild to moderate range, and organomegaly stabilized. Development remained normal. The older sibling had mild organomegaly and cardiac involvement by age 3 at T1, which remained mild with therapy. Motor development remained normal while learning difficulties present at onset of ERT persisted. Both siblings had progression of musculoskeletal symptoms, although severity remained milder in the younger sibling. Spinal radiographic findings for sibship 2 are shown in Fig. 3. Panels A and B are radiographs of the older sibling at age 8, after 5 years of ERT, showing significant scoliosis and other spinal deformities. Panels C and D are spinal radiographs of the younger sibling at age 10, after 10 years of ERT, showing the absence of spinal deformity.

#### Sibship 3

The older sibling had significant limitations in ADL, moderate musculoskeletal disease, and moderate to severe cognitive delay and delays in fine and gross motor development when ERT was initiated at 12 years of age. All existing symptoms persisted or progressed in this sibling except for improvement in organomegaly. The younger sibling began ERT at 8 months of age and after 8 years had no restrictions in ADL, no cognitive delays, and only mild musculoskeletal symptoms and mild to moderate ocular disease. Cardiac symptoms remained stable.

#### Sibship 4

Siblings had normal fine and gross motor development and were able to walk unassisted prior to the start of ERT. The older sibling also had no cardiac symptoms at T1. Cardiac disease was mild in both siblings at T2; other symptoms stabilized, except for progression of respiratory symptoms. Cognitive delays persisted in the older sibling, and developed in the younger. Both siblings had poor compliance (~60 %) with ERT, which may have impacted the symptom progression.

#### Sibship 5 (three siblings)

Two siblings initiated ERT before 5 years of age, one prior to the development of any symptoms, and one after mild cardiac symptoms, ocular disease, and organomegaly, and moderate delays in motor skills were apparent. Similar symptoms (except for the presence of moderate to severe organomegaly) were present in the oldest sibling who began ERT at 5.8 years of age. Additionally, this sibling presented with mild joint contractures at this time. At T2, there were improvements in organomegaly and motor function. Pulmonary function was at 30–40 % of normal at follow-up (no information available at T1). Ocular involvement and lower extremity joint symptoms progressed, but cardiac disease remained stable. In the sibling who initiated ERT at 2.5 years, organomegaly and motor function delays improved, cardiac symptoms remained stable, and mild joint contractures developed. The sibling who began ERT at <1 year of age remained healthy, with the exception of mild ocular and cardiac symptoms. Psychomotor development remained normal and there were no limitations in ADL. In both younger siblings, pulmonary function was between 70 and 94 % of normal at T2. In general, the severity of symptoms in the youngest sibling remained milder than those of the other siblings.

#### Sibship 6 (three siblings)

Two siblings initiated ERT at 11 and 13 years, respectively, and the youngest began ERT at 3 years of age. All three siblings had mild cardiac and musculoskeletal symptoms at T1. Cardiac symptoms remained mild at T2, and joint contractures progressed in all three siblings and were severe at T2. However, dysostosis multiplex progressed only in the older siblings to moderate to severe, and remained mild in the younger sibling. Pulmonary function was 45–50 % of normal in the two older siblings at T2, and 70–80 % of normal in the younger sibling. None of the siblings had restrictions in ADL.

#### Sibship 7

Both siblings began ERT prior to 5 years of age and had significant somatic, motor, and cognitive impairments prior to initiating therapy. After 5 years of ERT, cardiac symptoms stabilized, and musculoskeletal symptoms and motor development improved in the older sibling. Cardiac symptoms progressed in the younger sibling but remained mild. Corneal clouding progressed in both siblings. In the younger sibling who began both intensive physiotherapy and speech therapy at the age of 5, motor development remained normal, and cognitive skills and musculoskeletal disease improved.

#### Sibship 8

Both siblings had progression of cardiac symptoms at T2. Dysostosis multiplex stabilized in both siblings, and remained milder in the younger sibling. The younger sibling’s joint contractures progressed into the moderate range. While the older sibling had behavioral issues and required an aide for 1-on-1 schooling, the younger sibling had no such issues.

#### Sibship 9

Both siblings began ERT at a late age (14 and 8.6 years of age, respectively), and had similar symptom profiles at T1, with ocular, respiratory, cardiac, and musculoskeletal involvement. At T2, ocular and cardiac symptoms progressed from mild to moderate/severe in the older sibling. In addition, pulmonary function worsened, and contractures and lower extremity weakness progressed. In contrast, the younger sibling had stabilization of mild ocular symptoms, and cardiac disease remained less severe in the mild to moderate range. Both patients had stabilization of dysostosis multiplex and no progression of motor function delays.

**Fig. 4:**
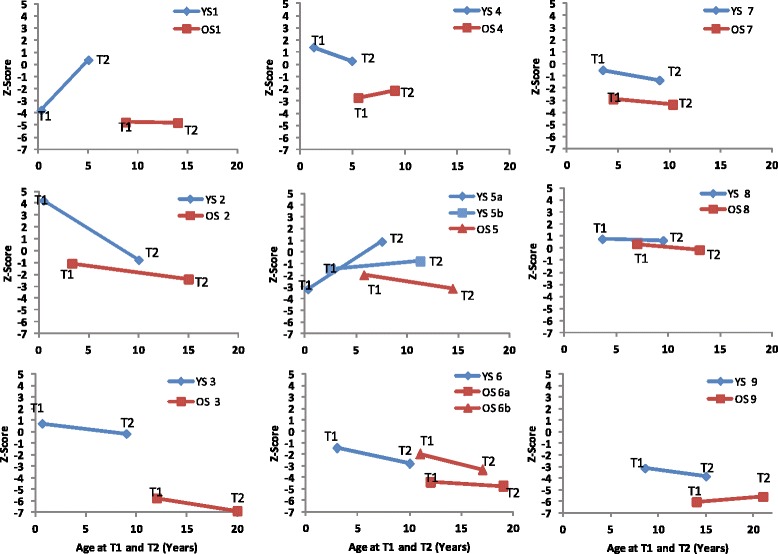
Z-scores for height/length at T1 (time of ERT initiation) and T2 (follow-up) for older (OS) and younger siblings (YS). Group A: younger siblings ERT onset ≤1 yr of age (sibships 1-5a; Group B: younger siblings ERT onset between 2 and 4 yrs (except sibship 9 at 8.5 yr) (sibships 5b through 9)

All older siblings except those from sibships 2 and 8 had Z-scores greater than two standard deviations below the mean (less than −2). At T2, Z-scores deviated further from the mean in the majority of the older siblings. Although remaining two standard deviations below the mean, in sibship 4, the Z-score improved from −3 to −2 and in sibship 9 improved from −6 to −5.6. In general, Z-scores for younger siblings at T2 were closer to sex- and age-matched means. For 6/9 of the younger siblings, Z-scores ranged from +0.88 to −1.4 at T2, and represented either improvements in height/length or no significant change from T1. For the younger siblings in sibships 6 and 9, the Z-scores at T2 were more negative than at T1 with height/length 2.5 or more standard deviations from the mean. Importantly, in all sibships (with the exception of sibship 6) the heights of the younger siblings at T2 were greater than their older siblings at comparable ages. Height/length Z-scores for all sibships at T1 and T2 are shown in Fig. 4.

## Discussion

The data collected from this multinational case series demonstrate the benefit of early ERT initiation in patients with attenuated MPS I. Laronidase is believed to stabilize but not improve cardiac and skeletal/connective tissue disease when pathological changes are already present at the start of treatment, and early ERT may help prevent the onset of disease features [8]. However, there are limited studies in young patients with clinically significant somatic symptoms prior to initiation of ERT. The pivotal laronidase clinical trial for MPS I enrolled patients 5 years of age and older [6]. A subsequent trial enrolled patients younger than 5 years of age and reported improved clinical status after 1 year of treatment with no safety concerns [5]. Previously-published single case studies in siblings with MPS I support the pre-symptomatic initiation of ERT to prevent or delay many clinical manifestations of the disease [9, 10]. The alteration of clinical disease progression has also been described in sibling pairs with MPS VI where early, pre-symptomatic initiation of ERT in the younger sibling resulted in prevention of symptoms, including bone deformities, and sustained benefits over 10 years of treatment [11–13]. Studies in animal models of MPS I also support early initiation of ERT. Laronidase treatment initiated soon after birth in affected dogs and mice improves outcomes and prevents cardiac and skeletal disease [14, 15]. Our comparison of disease progression following laronidase treatment in a series of older and younger siblings with MPS I lends additional support to the value of early ERT initiation.

Grouping sibships based on the age of ERT initiation in the younger sibling provided additional insight into the benefits of early treatment and the development of clinical features. In Group A (younger sibling initiated treatment ≤1.3 years of age), mild to severe symptoms were noted before treatment initiation in older siblings while symptoms were absent to mild in younger siblings. Most striking is the continued absence of clinical symptoms in the younger sibling from sibship 1 after nearly 5 years of ERT contrasted to the stabilization, but not reversal, of symptoms in the older sibling. Prominent differences were seen within other sibships in Group A for corneal opacity, organomegaly, dysostosis multiplex, and joint contractures. The extent of cardiac involvement was variable amongst siblings; however, improvement or stabilization of symptoms was noted following ERT. Younger siblings in Group A also tended to have fewer delays in motor and cognitive function, and less restriction in ADL.

For Group B (younger sibling initiated treatment between 2 and 4 years, with the exception of sibship 9), symptoms present in older siblings at T1 ranged from mild to severe. In younger siblings, symptoms generally ranged from absent to moderate, largely depending on the age treatment was initiated. At T2, symptoms noted in younger siblings at the time of ERT initiation either improved or stabilized, and only one developed symptoms classified as severe (sibship 6; joint contractures). Clinical differences were noted between older and younger siblings in Group B for organomegaly and dysostosis multiplex. Following treatment initiation, older siblings experienced improvements (especially in symptoms that were severe at baseline) or stabilization while younger siblings in this group experienced slower and less severe disease progression.

Short stature is a key clinical feature of MPS I. The rate of growth in children with MPS I is slower than in the healthy population, and differences in height between healthy and affected children increase with age [16]. Some studies have not found early ERT therapy to be associated with improved growth patterns in children with attenuated MPS I [16, 17]. However, a case study of three siblings reported stabilized or improved growth in two of three siblings who initiated laronidase therapy before 5 years of age [10]. In contrast, growth was further from the normal population mean in the third sibling who initiated laronidase therapy at age 6. Our data support the findings that ERT does not lead to improvement in growth. However, younger siblings remained taller than their older counterparts at a comparable age.

As a retrospective study, our analyses were limited by lack of control over the primary data collection, missing data, and issues with objective data analysis. While we recognize that the assessment of symptoms severity by physicians was subjective and could be influenced by sociocultural differences in medical practice, it is important to note that the patients were followed for most of their lives by the same physicians, who were able to compare symptoms in the siblings for a number of years and through various developmental stages. We believe this factor can limit the bias in assessing severity between siblings. Given the difficulties in conducting randomized controlled trials in the setting of a rare disease, and the geographical dispersion of patients with MPS I, we believe that comparing data between the siblings rather than across patients is an effective retrospective design. While comparing siblings is an insightful model to better understand the benefits of early treatment initiation in MPS I due to similar genetic backgrounds, there are a number of factors to consider. Phenotypic variability resulting from the effects of modifying polymorphisms is known to occur with some mutations [18], including the p.P533R homozygous mutation present in sibships 1 and 2. In general, the phenotypes between siblings within these sibships were similar. While we cannot totally exclude phenotypic differences as playing a role in the different clinical outcomes between siblings, in general we believe that there is a tendency for family group clusters for disease presentation in affected siblings, and that phenotypic differences are more pronounced between, rather than within, families.

Compliance with ERT is important in order to achieve maximal clinical effect [19]. In our case series, both siblings in sibship 4 had a reported ERT compliance rate of 60 %, and the younger sibling experienced progressive joint contractures and cognitive delays similar to the older sibling despite initiating therapy at an earlier age While cognitive function is not believed to be directly affected by ERT in patients with MPS I since laronidase does not cross the blood brain barrier, improvements in somatic disease following ERT may impact results of cognitive testing. Likewise, interventions including hearing aids, speech t herapy and physical therapy, as well as psychosocial assistance can optimize patient’s capacities and quality of life. Improvements in treatment outcomes were augmented in patients, such as those in sibship 7, who received supportive care along with ERT. The available data support the role of early or pre-symptomatic ERT initiation in slowing disease progression in patients with attenuated MPS I. Especially for disease manifestations that may be irreversible despite laronidase therapy, such as cardiac and musculoskeletal symptoms, early treatment is believed to be key in slowing the progression of these symptoms [7].

## Conclusion

These findings support early initiation of ERT with laronidase prior to the onset of symptoms in patients with attenuated MPS I in order to delay disease progression and/or prevent the development of severe clinical manifestations.
